# Botulinumtoxin Improves both Generic and Disease-Specific Quality of Life in Cervical Dystonia

**DOI:** 10.3389/fneur.2017.00561

**Published:** 2017-10-24

**Authors:** Daniel Weiss, Leonhard Hieber, Justine Sturm, Axel Börtlein, Ingo Mayr, Matthias Appy, Benedicta Kühnler, Joachim Buchthal, Christian Dippon, Guy Arnold, Tobias Wächter

**Affiliations:** ^1^Hertie-Institute for Clinical Brain Research, Department of Neurodegenerative Diseases, Tübingen, Germany; ^2^Department of Neurodegenerative Diseases, Centre of Neurology, University of Tübingen, Tübingen, Germany; ^3^Neurologische Klinik, Klinikum Stuttgart, Stuttgart, Germany; ^4^Klinik für Neurologie Sindelfingen, Krankenhaus Sindelfingen-Böblingen, Sindelfingen, Germany; ^5^Berufsausübungsgemeinschaft Dres. Matthias Appy, Wolfgang Molt, Prof. Arthur Melms und Kollegen, Stuttgart, Germany; ^6^Neurologische Gemeinschaftspraxis am Seelberg, Stuttgart, Germany; ^7^Abteilung für Neurologie, Reha-Zentrum Bad Gögging, Passauer Wolf, Bad Gögging, Germany

**Keywords:** cervical dystonia, segmental dystonia, blepharospasm, botulinumtoxin, quality of life

## Abstract

Botulinumtoxin injection (BoNT) into affected muscles is effective to improve motor symptoms of cervical dystonia (CD) by reducing muscle contraction and involuntary dystonic movement and posturing. However, the understanding of the effect on health-related quality of life (HR-QoL) and patient referral under HR-QoL aspects is incomplete. In this open-label clinical prospective observational study, we characterized the outcomes in CD (*n* = 159) from botulinumtoxin on both generic HR-QoL (EuroQol; EQ-5D-5L) and disease-specific HR-QoL [craniocervical dystonia questionnaire (CDQ-24)]. Additionally, we characterized motor and non-motor signs of dystonia including motor symptom improvement, depressive symptoms, pain, and sleep quality. We assessed patients at the end of a regular 3-month period from last injection (Timepoint1) and 4 weeks after the re-injection of BoNT (Timepoint2). We aimed to define outcomes on both generic and disease-specific HR-QoL and to evaluate predictors of therapeutic outcome in terms of stepwise multiple regression models. Patients with CD showed a robust improvement of both generic and disease-specific HR-QoL. Furthermore, motor and non-motor signs improved. Multiple regression analyses revealed that EQ-5D-5L and “satisfaction with health” (Fragen zur Lebenszufriedenheit-G) at Timepoint1 predicted treatment response on generic HR-QoL outcome (*R*^2^ = 0.284; *P* = 0.019). Similarly, CDQ-24 and Beck’s Depression inventory at Timepoint1 predicted the treatment response on disease-specific HR-QoL (*R*^2^ = 0.253; *P* = 0.026). Our study underscores both generic and disease-specific HR-QoL improvements in CD, and provides useful predictors on HR-QoL outcomes.

## Introduction

Health-related quality of life (HR-QoL) is impaired in cervical dystonia (CD) when compared with the healthy control population (1–5). CD includes abnormal posturing and involuntary movements of the head and neck. In this sense, a robust and well-reproduced motor effect has been demonstrated in CD (6, 7). However, HR-QoL does not linearly relate to motor response but incorporates multivariate modifiers including non-motor features (4). Thus, the understanding of HR-QoL effects from botulinumtoxin injection (BoNT) treatment is complex. HR-QoL in CD seems to be modulated by associated non-motor signs in particular (8). As such, anxiety was identified as predictor on HR-QoL costs in CD patients (9). This stands in accordance to a previous study that pointed to psychiatric comorbidity as a major determinant of disability in CD (8). Moreover, depressive symptoms are pronounced in CD (10, 11), and good BoNT responders yielded lower depression scores and lower dystonia motor severity (1). Moreover, self-efficacy was identified as most salient predictor in a non-motor model predicting self-perceived disability (4). Pain is a predominant feature in up to 75% of the patients with CD (12) and impacts HR-QoL. Nevertheless, such conclusions should be substantiated on larger studies and patient samples (13–15). This implies the need for additional insight and inclusion of larger and comprehensive data sets including assessments of motor and non-motor signs. In this sense, clinical studies on HR-QoL outcomes became a mainstay for therapy referral in wide fields of movement disorders, including dystonia and Parkinson’s disease (16–18). Moreover, longitudinal surveys may help to identify meaningful predictors, when approximating the potential effect of BoNT on HR-QoL outcomes in patients with CD.

In this open-label clinical prospective observational study, we address this need by characterizing outcomes on both generic and disease-specific HR-QoL from repetitive BoNTs in a cohort of 159 CD patients. We corroborated these HR-QoL assessments by an in-depth evaluation of motor and non-motor aspects related to CD. The latter included assessments on dystonia motor severity, head tremor, different life satisfaction domains, depressive symptoms, pain, and sleep quality. To this end, we characterized these outcome variables at the end of a regular 3-month washout period from last injection and 4 weeks after re-injection of BoNT, which is considered as time point yielding the peak maximum of the clinical effect (7, 19, 20). We used stepwise multiple regression models to predict treatment response of both generic and disease-specific HR-QoL.

## Materials and Methods

### Patients and Study Design

We conducted comprehensive assessments in CD patients treated with BoNT (*n* = 159; detailed patient characteristics in Table 1). The vast majority of these patients received ongoing treatment with repeated injections in 3-month intervals, whereas only four patients received first-time injections of BoNT. We performed this clinical study as an open-label observational clinical study. Although such a study protocol is less standardized when compared with randomized-controlled trials, it bears some strengths, since the cohort is generally more representative (since less selected) for daily life clinical practice, and therefore valuable to complement findings from controlled trials. We recorded generic [EuroQol (EQ)-5D-5L] and disease-specific [craniocervical dystonia questionnaire (CDQ-24)] HR-QoL as dependent variables as well as further clinical scores (i) at the end of a 3-month interval from the last BoNT injection (prior to first injection in first-time recipients) (Timepoint1). This is in keeping with previous considerations, suggesting that the effects of BoNT on HR-QoL will be diminished 12 weeks after injection (2, 21). As follow-up assessment, we re-evaluated patients 4 weeks after injection (Timepoint2) reflecting the time point of expected optimal BoNT efficacy. Similarly, the EQ-5D-5L (used for HR-QoL assessments in this study) was suggested to reflect the subject’s situation at the time of completion (22) and makes no attempt to recall the health status over the preceding days or weeks.

**Table 1:** Characteristics of patients with cervical dystonia (CD).

	CD
Number of patients	159
Age (mean ± SD)	58.1 ± 12.0
Male: female	57: 102
Age at onset	43.7 ± 14.8
Disease duration	14.6 ± 11.5
Repeated injections	155
First-time injection	4
Dystonic head tremor	59
BoTN preparations (in units)	
Dysport	709 ± 278
Botox	176 ± 78
Xeomin	169 ± 85
Neurobloc	7,500

Patients were enrolled at the clinics of the neurological centers and private offices listed below. BoNT treatment was performed by neurologists with at least 5 years of experience in BoNT treatment. Inclusion criteria were the diagnosis of CD and age >18 years. We excluded patients treated with concomitant medications potentially interacting with BoNT treatment, such as oral anticoagulants (e.g., phenprocoumon) or aminoglycoside antibiotics. The local Ethics committee of Tübingen University (516/2011BO2) and of the Landesärztekammer Baden-Württemberg approved the study, and patients participated after written informed consent.

Patients were included, treated, and followed at the Centre for Neurology, Department for Neurodegenerative Diseases, Tübingen University, the Centre for Neurology Stuttgart Bürgerhospital, Centre of Neurology Sindelfingen, Neurology Practice “Dr. Appy & Molt” Stuttgart, and Neurology Practice “am Seelberg” Stuttgart.

Patients were treated with BoNT A (Dysport^®^, Botox^®^, and Xeomin^®^) or BoNT B (Neurobloc^®^) (Table 1).

### Assessments

As main interest, we analyzed outcomes on generic and disease-specific HR-QoL. We assessed generic HR-QoL in terms of the EQ self-rating scale. As first part, the EQ-5D-5L comprises five questions on subjectively perceived mobility, self-care, usual activities, pain/discomfort, and anxiety/depression. Patients reported on a five-point scale whether they have no problems (1), slight problems (2), moderate problems (3), severe problems (4), or extreme problems (5). The second part EQ-VAS is a vertical 20 cm visual analog scale from 0 to 100 with steps of one in order to assess the self-rated momentary health state (0 indicating worst possible state and 100 indicating best possible state). Disease-specific HR-QoL was analyzed with the CDQ-24 which is a 24-item version, where is item is self-rated and scored along the answers “never,” “occasionally,” “sometime,” “often,” or “always.”

Further, we recorded the questionnaire on life satisfaction validated in German language [i.e., “Fragen zur Lebenszufriedenheit” (FLZ)] including the modules on: “general life satisfaction” (FLZ-A), satisfaction with health (FLZ-G), satisfaction with movement disorder (FLZ-BS). Further, Beck’s depression inventory (BDI), subjective rating of perceived severity in both dystonia and head tremor (1–10), average and maximal pain during the last 7 days assessed as visual analog scale, Clinical Global Impression Scale, and Pittsburgh sleep quality index were assessed. Additionally, we report on the CDQ-24 in patients with CD and segmental dystonia (23). We obtained all scores in German language. We performed the study and all statistical analyses with exploratory intent.

### Statistical Analyses

We conducted non-parametric Wilcoxon tests with two-sided significance level of *P* < 0.05 (SPSS, Version 23) comparing the scores at Timepoint1 vs. Timepoint2. We corrected for false positives with the false discovery rate (24).

For outcome predictions (expressed as difference Timepoint2 − Timepoint1 in either generic or disease-specific HR-Qol measures), we used independent variables at Timepoint1 and integrated these scores into a stepwise multiple regression model. Outcomes on HR-QoL were treated as dependent variables, i.e., EQ-5D-5L for generic HR-QoL and CDQ-24 for disease-specific HR-QoL. Additional statistical diagnostics on multiple linear regression models included: independence of errors in terms of Durban Watson tests; linear relationships between independent and dependent variables integrated into the stepwise model; verification of homoscedasticity; exclusion of collinearity as reason for improved predictive value when adding consecutive variables to the prediction model; and normal distribution of the residuals.

## Results

Cervical dystonia patients showed improved generic HR-QoL in both EQ-5D-5L (*P* = 0.017) and EQ-VAS (*P* < 0.001) at Timepoint2 compared with Timepoint1. Similarly, disease-specific HR-QoL improved in terms of CDQ-24 (*P* < 0.001). These improvements in HR-QoL were corroborated by improvements in life satisfaction in the movement disorders domain. Motor symptoms in terms of dystonic posture and head tremor improved as well. As non-motor domains both “pain maxima” and “mean pain,” as well as depressive symptoms and sleep quality improved. Statistical comparisons of Timepoint1 and Timepoint2 are given as Table 2. Next, we aimed to predict the improvement of both generic and disease-specific HR-QoL from independent variables at Timepoint1. Therefore, we conducted stepwise multiple regressions, and tested whether the scores at Timepoint1 would predict the HR-Qol improvement expressed as difference between Timepoint2 and Timepoint1.

**Table 2:** Outcome in cervical dystonia (CD).

	Timepoint1 (mean ± SD)	Timepoint2 (mean ± SD)	*z*-Value	*P*-value
EQ-5D-1	3.81 ± 3.23	3.31 ± 3.05	−2.395	0.017*
EQ-5D-2	60.41 ± 22.14	69.39 ± 18.44	−4.500	0.000*
CDQ-24	31.52 ± 18.80	27.58 ± 17.91	−3.921	0.000*
FLZ-A	47.16 ± 33.89	44.96 ± 32.78	−0.657	0.511
FLZ-G	39.68 ± 35.80	43.68 ± 38.34	−1.989	0.047
FLZ-BS	70.39 ± 57.10	80.27 ± 57.10	−3.126	0.002*
BDI	10.23 ± 7.32	9.41 ± 6.78	−2.675	0.007*
Dystonia (1–10)	6.27 ± 6.92	4.00 ± 2.27	−6.451	0.000*
Head tremor (1–10)	4.70 ± 3.17	3.21 ± 2.53	−5.775	0.000*
Pain (maximum)	3.90 ± 3.11	3.40 ± 2.76	−2.613	0.009*
Pain (mean)	3.33 ± 2.76	2.92 ± 2.40	−2.718	0.007*
PSQI	6.71 ± 3.75	6.20 ± 3.40	−2.377	0.017*

*CD (*n* = 159). Non-parametric Wilcoxon tests were conducted on the two-sided significance level of *P* < 0.05. Significance level was adjusted for multiple comparisons using the false discovery rate*.

*Significance was decided on two-tailed P < 0.05 as is indicated in the legend.

**Figure 1:**
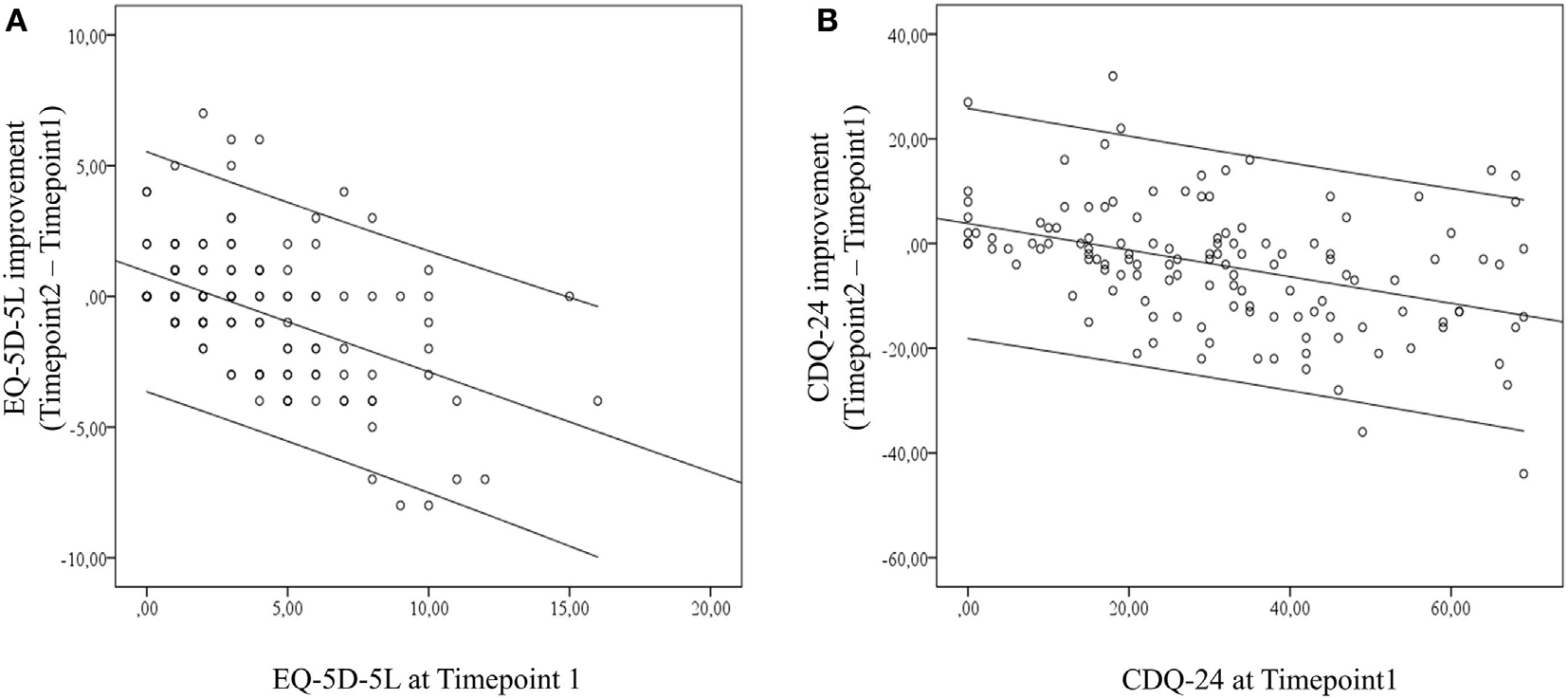
Univariate correlations of **(A)** generic health-related quality of life (HR-QoL) outcome and EuroQol-5D-5L at Timepoint1 and of **(B)** disease-specific HR-QoL outcome and craniocervical dystonia questionnaire at Timepoint1. Both scatter plots point to the relation of self-perceived HR-QoL impairments at Timepoint1 and treatment effect. Error indicators are given as 95% confidence limit.

Improvement on generic HR-QoL from BoNT in terms of the EQ-5D-5L was predicted by EQ-5D-5L and “satisfaction with health” (FLZ-G) at Timepoint1 (*R*^2^ = 0.284; adjusted *R*^2^ = 0.270; *P* = 0.019). Significant univariate correlations of generic HR-QoL improvement with independent variables included EQ-5D-5L, EQ-VAS, satisfaction with health (FLZ-G), satisfaction with movement disorder (FLZ-BS), dystonia motor symptom severity, pain maxima, and sleep quality (Table 3). The univariate correlation of EQ-5D-5L at Timepoint1 with treatment outcome on EQ-5D-5L indicates that the amount of self-perceived generic HR-QoL impairment is critical for treatment outcome (Figure 1A).

**Table 3:** Multiple regressions to predict of generic or disease-specific health-related quality of life (HR-QoL).

Independent variables at Timepoint1	Prediction of EQ-5D-5L improvement (generic)	Prediction of CDQ-24 improvement (disease-specific)
EQ-5D-5L	*R* = −0.494; *P* < 0.001*	*R* = −0.167; *P* = 0.046
EQ-VAS	*R* = 0.208; *P* = 0.018	*R* = 0.266; *P* = 0.003
CDQ-24	*R* = −0.150; *P* = 0.065	*R* = −0.463; *P* < 0.001*
FLZ-A	*R* = 0.074; *P* = 0.228	*R* = 0.029; *P* = 0.388
FLZ-G	*R* = 0.163; *P* = 0.049*	*R* = 0.100; *P* = 0.158
FLZ-BS	*R* = 0.210; *P* = 0.017	*R* = 0.164; *P* = 0.049
BDI	*R* = −0.125; *P* = 0.105	*R* = −0.212; *P* = 0.016*
Dystonia	*R* = −0.224; *P* = 0.012	*R* = −0.280; *P* = 0.002
Tremor	*R* = −0.048; *P* = 0.315	*R* = −0.179; *P* = 0.036
Pain intensity (max)	*R* = −0.165; *P* = 0.048	*R* = −0.226; *P* = 0.011
Pain intensity (mean)	*R* = −0.159; *P* = 0.055	*R* = −0.126; *P* = 0.104
Sleep quality (PSQI)	*R* = −0.218; *P* = 0.014	*R* = −0.146; *P* = 0.071

Predictions were made on improvement of both generic [EuroQol (EQ)-5D-5L] and disease-specific [craniocervical dystonia questionnaire (CDQ-24)] HR-QoL difference scores between Timepoint2 and Timepoint1 as dependent variables. Predictions were made from independent variables at Timepoint1; Pearson correlations are shown between the dependent variable and the independent predictor variables. Improvement on generic HR-QoL from botulinumtoxin injection in terms of the EQ-5D-5L was predicted by EQ-5D-5L and “satisfaction with health” [Fragen zur Lebenszufriedenheit (FLZ)-G] at Timepoint1 (R^2^ = 0.284; adjusted R^2^ = 0.270; P = 0.019). Disease-specific HR-QoL improvement was best predicted by CDQ-24 and Beck’s depression inventory (BDI) at Timepoint1 (R^2^ = 0.253; adjusted R^2^ = 0.238; P < 0.026). Independent variables used for the stepwise multiple regression model are marked with asterisks (*).

Disease-specific HR-QoL improvement was best predicted by CDQ-24 and BDI at Timepoint1 (*R*^2^ = 0.253; adjusted *R*^2^ = 0.238; *P* < 0.026). Significant univariate correlations of disease-specific HR-QoL improvement included CDQ-24, EQ-5D-5L, EQ-VAS, satisfaction with movement disorder (FLZ-BS), BDI, dystonia motor symptom severity, head tremor, and pain maxima (Table 3). The univariate correlation of CDQ-24 at Timepoint1 with treatment outcome on CDQ-24 indicates that the amount of self-perceived disease-related HR-QoL impairment is critical for treatment outcome (Figure 1B).

## Discussion

In this observational open-label clinical study, we found that BoNT therapy was effective on both generic and disease-specific HR-QoL. This was corroborated by improvements of motor symptoms and non-motor domains, namely life satisfaction, depressive symptoms, pain, and sleep quality.

In order to stratify patient outcomes from BoNT under HR-QoL aspects, we aimed to predict the treatment response of this longitudinal cohort on both generic and disease-specific HR-QoL improvements from Timepoint1 scores. Generic HR-QoL improvements were predicted by stronger EQ-5D-5L and “satisfaction with health” impairments at Timepoint1, whereas other motor and non-motor assessments at Timepoint1 did not add predictive value. Disease-specific HR-QoL showed stronger improvements upon higher CDQ-24 impairments and higher BDI scores at Timepoint1. Interestingly, a broad set of motor and non-motor variables at Timepoint1 was correlated to both generic and disease-specific HR-QoL outcomes, supporting that both generic and disease-specific HR-QoL are complex constructs associating with a highly dimensional set of variables in accordance to previous findings (1, 8, 9). However, not all of these associated variables—although clinically meaningful—were valuable to inform on treatment prognosis. Hence, the present study may yield its largest value in disentangling meaningful surrogates for response prediction under ongoing BoNT treatment.

Our findings are in accordance with previous literature as both motor and non-motor symptoms improved with BoNT treatment in CD and, therefore, probably contribute to improvements in HR-Qol. However, the pure motor symptom severity as well as depressive symptoms that were associated with treatment response did surprisingly not predict generic HR-QoL improvement, although both variables were associated with HR-Qol costs previously (9). Interestingly, it was more the self-perceived HR-QoL cost at Timepoint1 that was most indicative for treatment response, and slightly larger predictive accuracy was achieved when adding “satisfaction with health” (FLZ-G) to the stepwise multiple regression model. Similarly, treatment outcome in disease-specific HR-QoL was best predicted by CDQ-24 at Timepoint1, and adding BDI to the stepwise regression model slightly increased diagnostic accuracy. Together, both generic and disease-related HR-QoL outcomes should be evaluated in the context of self-perceived impairments in HR-QoL domains when administering BoNT treatment—in other words, a critical threshold of self-perceived impairment at the end of a 3-month re-injection interval may indicate a higher chance of treatment success in an individual patient. This may be a very useful complementary tool to re-evaluate effects under ongoing BoNT treatment, i.e., to re-evaluate at a certain time point of a long-term treatment intervals whether BoNT exerts the desired effect in an individual patient.

In particular, in previous studies depressive symptoms and pain seemed to have a stronger effect on HR-QoL costs when compared with the present study (4, 9–11). One study indicated that higher generic HR-QoL costs were predicted by depressive symptoms in a cross-sectional cohort of CD patients (9). Notably, however, our study design was different and aimed to quantify and predict the treatment response after BoNT (re-)injection, and to establish a regression model for outcome prediction, which is presently not available to the best to our knowledge. In this context, as major finding from our study, depressive symptoms or pain did not indicate the potential response of HR-QoL to repeated BoNT injections. This is not to say that depressive symptoms or pain would not impact self-perceived HR-QoL measures, however, indicate distinct aspects of a much more complex and multidimensional construct influenced by many intra- and interpersonal, psychosocial, and environmental factors (4) as can be seen from the wide-spread correlations of both generic and disease-specific HR-QoL measures in our study.

### Methodological Considerations

We suggest considering with caution some natural limitations of open-label observational studies like this. Most of our patients were under chronic treatment with BoNT and assessed at the end of a regular 3-month re-injection interval. Therefore, we cannot exclude underestimation of the true effect size, i.e., we cannot be entirely sure that the BoNT effect on HR-QoL was fully washed-out at the end of a 3-month interval prior to re-injection. However, we did not consider longer washout periods for clinical and ethical reasons. Nevertheless, our study pointed to clinically relevant effects after a period of 3 months from last injection and corroborative evidence exists that therapeutic effects of BoNT on HR-QoL may dissipate within a 3-month period (2).

Further, we mainly considered patients under ongoing BoNT therapy. As consequence, our study would not reflect patients with unsatisfactory response and early discontinuation of BoNT injections. To control for these aspects in future studies, follow-up assessments on first-time injected patients might be considered. Instead, however, rapid establishment of a fully effective and well-tolerated individualized injection scheme would be challenging and potentially limiting in such an alternative scenario. With respect to generalizability, we kept inclusion and exclusion criteria widely open. Therefore, as major strength, our study represents findings from a standard of clinical care “real-world” setting. Instead, more stringently selected cohorts from randomized-controlled clinical trials are often limited in generalizing to daily life clinical care.

Together, we suggest to consider the findings from this observational study supportive for standard of clinical care settings, when deciding on BoNT efficacy on HR-QoL in patients under ongoing BoNT therapy. We trust that the findings from this study may help to construct larger studies to stratify patient referral for BoNT treatment in CD patients under HR-QoL considerations.

## Ethics Statement

The study was approved by the Ethics committee of Tübingen University and Landesärztekammer Baden-Württemberg. All patients participated with written informed consent.

## Author Contributions

Conception and design of the study: AB, IM, MA, BK, JB, CD, GA, and TW; acquisition of data including data analysis: DW, LH, JS, AB, IM, MA, BK, JB, CD, GA, and TW; first draft: DW and TW; drafting the article: DW, LH, JS, AB, IM, MA, BK, JB, CD, GA, and TW; revising it critically for important intellectual content and final approval of the version to be submitted: all the authors.

## Conflict of Interest Statement

None of the authors reports personal conflict of interest. The study received financial support from Merz Pharmaceuticals.
